# Malignant Peripheral Nerve Sheath Tumour of the Forearm Presenting as Foreign Body

**DOI:** 10.7759/cureus.15229

**Published:** 2021-05-25

**Authors:** Georgios Arealis, Konstantinos Kazamias, Khalid Malik Tabassum, Neil Ashwood

**Affiliations:** 1 Trauma and Orthopaedics, East Kent Hospitals University NHS Foundation Trust, Canterbury, GBR; 2 Trauma and Orthopaedics, Royal Surrey County Hospital, Guildford, GBR; 3 Trauma and Orthopaedics, University Hospitals of Derby and Burton NHS Foundation Trust, Derby, GBR

**Keywords:** malignant peripheral nerve sheath tumour, mpnst, foreign bodies, histology, soft tissue sarcoma

## Abstract

Malignant peripheral nerve sheath tumour (MPNST) is a rare form of soft tissue sarcoma that arises from peripheral nerves, accounting for less than 5% of cases. MPNST most commonly affects trunk and extremities, and It is commonly associated with neurofibromatosis type 1 (NF1) (40%-50%). We present a case of MPNST in a 52-year-old man with history of well-controlled epilepsy. He presented with a painful and erythematous mass in his left forearm, which was initially diagnosed as an abscess secondary to retained foreign bodies. Despite incision and drainage, he experienced recurrence of this mass two months later. Subsequent debridement, biopsy and histology revealed a high-grade MPNST. This prompted a referral to the regional sarcoma unit. Unfortunately, repeat scans demonstrated rapid progression of disease into the anterior forearm compartment and bony invasion. Despite radiotherapy, the tumour metastasised to his lungs. After undergoing palliative chemotherapy, unfortunately, the patient survived only 14 months from the initial presentation. Our study affirms that all resected tissues should be sent for histological confirmation of the suspected diagnosis. When intraoperative findings do not correlate with the initial presentation, the clinician should have a high index of suspicion for potential malignancy. Finally, it is essential that all patients with soft tissue sarcoma should be referred to the specialist regional soft tissue sarcoma service, to be managed by a specialist sarcoma multidisciplinary team according to guidelines.

## Introduction

Malignant peripheral nerve sheath tumour (MPNST) is a rare form of soft tissue sarcoma that arises from peripheral nerves [1], accounting for less than 5% of cases [2] and with an incidence of 0.001% in the general population [3]. It is commonly associated with neurofibromatosis type 1 (NF1) (40%-50%) [1]. MPNST is known for its aggressive malignant potential and commonly metastasises to the lungs. Unlike our case report, most malignant sarcomas have a very rapid progression and this makes them easier to identify [4]. As the mainstay of treatment is radical local resection; late diagnosis has an unfavorable prognosis [5].

We report a case of MPNST of the forearm in a patient, without a history of NF1, presenting as an abscess, related to a retained foreign body, with a background of trauma to the same forearm.

## Case presentation

A 52-year-old, manual labourer with a previous history of well-controlled epilepsy presented to the Accident and Emergency department with pain and swelling to the proximal dorsal aspect of his left forearm. He sustained a fall on his left elbow six months prior to presentation that resulted in superficial wounds with some associated debris and gravel from the rough ground.

**Figure 1:**
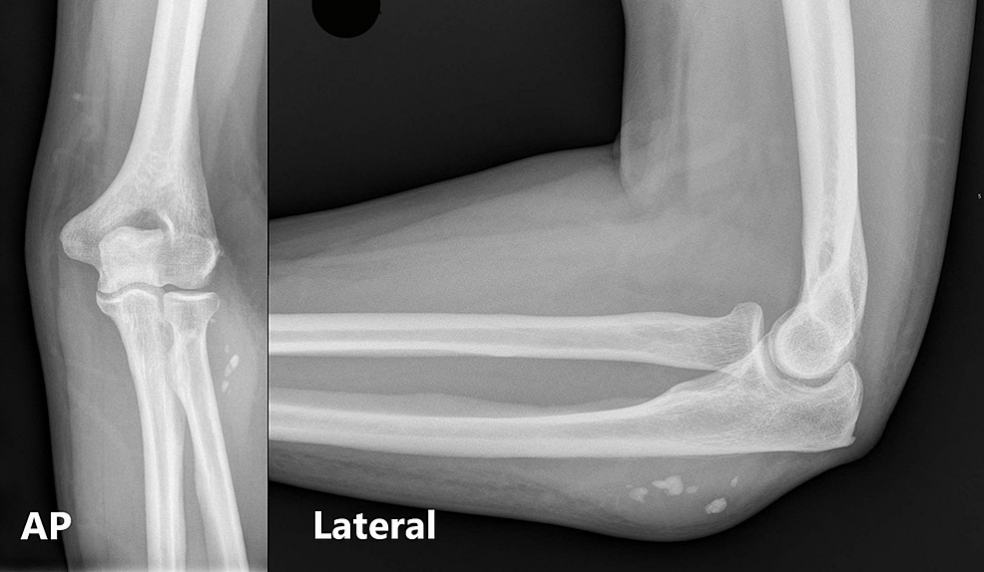
X-rays of the elbow on initial presentation with 'foreign bodies' present

**Figure 2:**
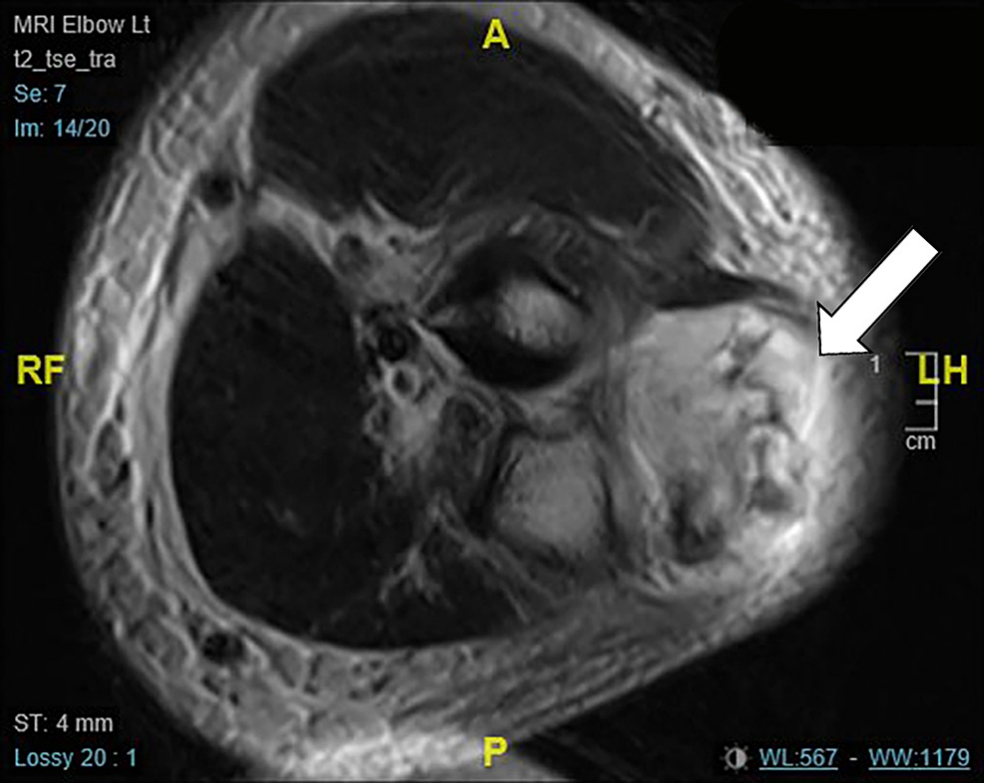
Initial MRI, white arrow points to the reported collection around 'foreign bodies'

On examination, he was noted to have a 5 x 8 cm raised, firm, tender, erythematous mass. Neurovascular examination was normal. Plain radiograph of his forearm revealed several calcified areas that were considered to be foreign bodies, due to the previous injury, each measuring <1 cm (Figure 1). Clinical examination and the presence of raised inflammatory markers (white cell count 8.8, C-reactive protein 172) indicated a provisional diagnosis of an abscess related to retained foreign bodies. He was admitted for elevation, intravenous antibiotics and an urgent inpatient MRI. The MRI reported an extra-articular 4 cm area of collection around the foreign bodies, in the proximal dorsal compartment of his forearm (Figure 2). He subsequently underwent incision and drainage in the operating theatre and was discharged with oral antibiotics. At two weeks clinic follow up, his wound had healed with no postoperative complications.

He re-presented to the clinic two months later with recurrence of pain and swelling. On this occasion, the painful mass was not erythematous. He was listed for “removal of foreign bodies and debridement” on an urgent trauma list. Intra-operatively, the findings were not consistent with recurrent infection. No pus was identified, and the extensor muscle mass appeared abnormal, with discoloration, scar, and necrotic areas. Due to the intraoperative findings, an area of 65 x 50 x 30 mm, that was believed to contain the foreign bodies, was excised. Complete removal of the foreign bodies was confirmed with fluoroscopy. As per standard routine for all resected tissues, the mass removed was sent for microbiology and histology.

Histology identified sections of synovium infiltrated by a high-grade sarcoma. The tumour had a spindle cell component, where the spindle cells were arranged in short, intervening fascicles, focally with storiform pattern. The spindle cells were surrounded by fibrous stroma. In other parts, the tumour cells had more rounded nuclei and the stroma appeared looser and myxo-oedematous. Small areas with rhabdoid morphology and haemangiopericytoma-like pattern were also noted. Necrosis was present. Mitoses, including atypical mitoses, were easily identified.

On immunostaining, the tumour cells were positive for vimentin and B cell lymphoma‐2 (BCL‐2). There was also positivity for CD99 and CD34, mainly in the spindle cell component. A few tumour cells were positive for cytokeratin 7 (CK7) and, in the rhabdoid looking area, for desmin. Stain for epithelial membrane antigen (EMA) was equivocal. Stains for Wilms tumour suppressor gene (WT1), S100, Melan A, and CK18 were negative.

The patient and histology were urgently referred to the local regional sarcoma unit (Royal Marsden NHS Foundation Trust, UK). Further histology found CD56 to be positive whereas myogenin, MyoD1, SOX1 STAT6, GFAP and MUC4 were negative. Additional molecular genetics were performed at the Royal Orthopaedic Hospital, UK. Both fluorescence in-situ hybridisation (FISH) and polymerase chain reaction (PCR) provided no support for synovial sarcoma, common variants of Ewing sarcoma or BCOR-CCNB3 tumour. Therefore, a final histological diagnosis of MPNST with rhabdomyoblastic differentiation was established.

**Figure 3:**
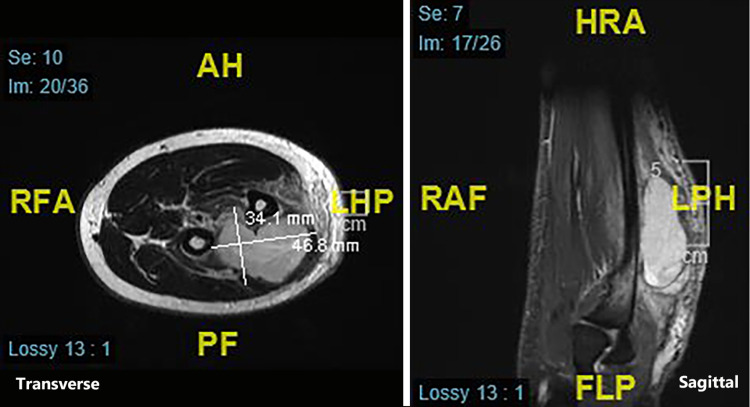
Repeat MRI scan with significant progression of the malignant peripheral nerve sheath tumour (MPNST) evident

Following this, an initial staging CT did not reveal evidence of metastatic disease, but a repeated MRI scan showed significant disease progression with extension into the deep extensor compartment, interosseous membrane, and anterior compartment of the forearm (Figure 3). Due to the location of the primary tumour, early bony changes and high-grade histological subtype, it was deemed as surgically inoperable without amputation. Initial treatment plan was to start radiotherapy with surveillance imaging and discussion for consideration for amputation in the future six weeks after the diagnosis was established.

The subsequent surveillance imaging revealed metastatic lung and brain disease. The patient was commenced on palliative chemotherapy with ifosfamide and doxorubicin and received six cycles of chemotherapy. Unfortunately, the disease progressed rapidly, and the patient survived only 14 months from initial presentation.

## Discussion

Soft tissue sarcomas account for about 1% of malignant tumours and MPNST account for 5%-10% of all soft tissue sarcomas [6]. Due to its uncommon nature, there is a paucity of data in the literature regarding prognostic factors and long-term outcomes [6]. MPNST is more common in patients with NF1, with an incidence of 4% to 13%, but are rare in the general population with an incidence of 0.001% [3,7]. The most common age of presentation is between 20 and 50 years. Extremities, more upper than lower limb, are the usual locations (45%); the trunk (34%) and head and neck (19%) follow [5]. Uncommon areas of presentation like scalp [8], abdomen [9], including bladder [10], have been reported, more often in NF1 patients [7]. Our patient’s age and presentation in the forearm are consistent with the common presentation of MPNST but as he had no history of NF1; there was very little suspicion that he could present with sarcoma and the obvious abscess due to injury was the initial, but mistaken, diagnosis.

MPNST, as most sarcomas, progresses rapidly, has a poor prognosis and vague nonspecific symptomatology. Our case report confirms those characteristics, as our patient presented with a vague history of trauma and abscess in the forearm that caused the initial misdiagnosis. Once MPNST was confirmed, and despite treatment at a specialist centre, survival was only 14 months, from the initial presentation.

Malignant tumours should always be included in the differential diagnosis of a soft tissue mass, especially in atypical cases. A low-threshold approach towards sending tissue samples for histopathology should be applied by clinicians. Conventional imaging techniques are not helpful in the diagnosis of MPNST. This is confirmed by our case where the initial MRI was reported as an abscess in the forearm. A biopsy followed by histopathology is diagnostic but requires a series of specialist tests and experience. Therefore, prompt diagnosis and referral to a sarcoma unit may help gain early local control with radical resection and radiotherapy. However, this does not prevent the development of distal metastasis. Any patient with soft tissue sarcoma should be referred to the specialist regional soft tissue sarcoma service, to be managed by a sarcoma multidisciplinary team according to guidelines [11].

## Conclusions

We report a rare case of a high-grade MPNST that was initially misdiagnosed due to presentation as a foreign body infection. Following resection, and histological confirmation of the diagnosis, treatment, according to oncology guidelines, was commenced but the patient survived only 14 months from the initial presentation. Despite appearing benign, at the initial presentation, it would be advisable to proceed with a complete pre-operative radiological investigation of such masses, with vague clinical history, especially when areas of calcifications are seen in the plain X-rays. The possibility of a malignant soft tissue tumour, with a bad prognosis, has to be kept in mind even in relatively straightforward cases, as a pre-operative staging could provide scope for better planning and management. Our study affirms that all resected tissues should be sent for histological confirmation of the suspected diagnosis. When intraoperative findings do not correlate with the initial presentation, the clinician should have a high index of suspicion for potential malignancy. Finally, early referral to a specialist regional soft tissue sarcoma unit is important. It increases the chances of survival, as the patient can be managed by a sarcoma multidisciplinary team according to international guidelines.

## References

[1] Baehring JM, Betensky RA, Batchelor TT (2003). Malignant peripheral nerve sheath tumor: the clinical spectrum and outcome of treatment. Neurology.

[2] DeWitt JC, Mock A, Louis DN (2017). The 2016 WHO classification of central nervous system tumors: what neurologists need to know. Curr Opin Neurol.

[3] Ducatman BS, Scheithauer BW, Piepgras DG, Reiman HM, Ilstrup DM (1986). Malignant peripheral nerve sheath tumors. A clinicopathologic study of 120 cases. Cancer.

[4] Abdel Al S, Abou Chaar MK, Asha W, Al-Najjar H, Al-Hussaini M (2020). Fungating malignant peripheral nerve sheath tumor arising from a slow-growing mass in the forearm: a case report and review of the literature. J Med Case Rep.

[5] Stucky CC, Johnson KN, Gray RJ, Pockaj BA, Ocal IT, Rose PS, Wasif N (2012). Malignant peripheral nerve sheath tumors (MPNST): the Mayo Clinic experience. Ann Surg Oncol.

[6] Zou C, Smith KD, Liu J (2009). Clinical, pathological, and molecular variables predictive of malignant peripheral nerve sheath tumor outcome. Ann Surg.

[7] Prudner BC, Ball T, Rathore R, Hirbe AC (2020). Diagnosis and management of malignant peripheral nerve sheath tumors: current practice and future perspectives. Neurooncol Adv.

[8] Firdaus M, Gill AS, Mukarramah DA, Andriani R, Sari L, Cahyanti D, Faried A (2018). Malignant peripheral nerve sheath tumor of the scalp: two rare case reports. Surg Neurol Int.

[9] Dare AJ, Gupta AA, Thipphavong S, Miettinen M, Gladdy RA (2020). Abdominal neoplastic manifestations of neurofibromatosis type 1. Neurooncol Adv.

[10] Petracco G, Patriarca C, Spasciani R, Parafioriti A (2019). Malignant peripheral nerve sheath tumor of the bladder: a case report. Pathologica.

[11] Dangoor A, Seddon B, Gerrand C, Grimer R, Whelan J, Judson I (2016). UK guidelines for the management of soft tissue sarcomas. Clin Sarcoma Res.

